# Reverse‐Bayes methods for evidence assessment and research synthesis

**DOI:** 10.1002/jrsm.1538

**Published:** 2021-12-30

**Authors:** Leonhard Held, Robert Matthews, Manuela Ott, Samuel Pawel

**Affiliations:** ^1^ Department of Biostatistics University of Zurich Zurich Switzerland; ^2^ Department of Mathematics Aston University Birmingham UK; ^3^ Data Team, Swiss National Science Foundation Bern Switzerland

**Keywords:** Analysis of Credibility, Bayes factor, false positive risk, meta‐analysis, prior‐data conflict, Reverse‐Bayes

## Abstract

It is now widely accepted that the standard inferential toolkit used by the scientific research community—null‐hypothesis significance testing (NHST)—is not fit for purpose. Yet despite the threat posed to the scientific enterprise, there is no agreement concerning alternative approaches for evidence assessment. This lack of consensus reflects long‐standing issues concerning Bayesian methods, the principal alternative to NHST. We report on recent work that builds on an approach to inference put forward over 70 years ago to address the well‐known “Problem of Priors” in Bayesian analysis, by reversing the conventional prior‐likelihood‐posterior (“forward”) use of Bayes' theorem. Such Reverse‐Bayes analysis allows priors to be deduced from the likelihood by requiring that the posterior achieve a specified level of credibility. We summarise the technical underpinning of this approach, and show how it opens up new approaches to common inferential challenges, such as assessing the credibility of scientific findings, setting them in appropriate context, estimating the probability of successful replications, and extracting more insight from NHST while reducing the risk of misinterpretation. We argue that Reverse‐Bayes methods have a key role to play in making Bayesian methods more accessible and attractive for evidence assessment and research synthesis. As a running example we consider a recently published meta‐analysis from several randomised controlled trials (RCTs) investigating the association between corticosteroids and mortality in hospitalised patients with COVID‐19.

**Highlights**

**What is already known?**

Standard methods of statistical inference have led to a crisis in the interpretation of research findings. The adoption of standard Bayesian methods is hampered by the necessary specification of a prior level of belief.

**What is new?**

Reverse‐Bayes methods open up new inferential tools of practical value for evidence assessment and research synthesis.

**Potential impact for RSM readers**

Reverse‐Bayes methodology enables researchers to extract new insights from summary measures, to assess the credibility of scientific findings and to reduce the risk of misinterpretation.

## 1 INTRODUCTION: THE ORIGIN OF REVERSE‐BAYES METHODS

“We can make judgments of initial probabilities and infer final ones, or we can equally make judgments of final ones and infer initial ones by *Bayes's theorem in reverse*.” I. J. Good^1^ ^(p29)^

There is now a common consensus that the most widely‐used methods of statistical inference have led to a crisis in both the interpretation of research findings and their replication.^2^, ^3^ At the same time, there is a lack of consensus on how to address the challenge,^4^ as highlighted by the plethora of alternative techniques to null‐hypothesis significance testing now being put forward, see for example Wasserstein et al.^5^ and the references therein. Especially striking is the relative dearth of alternatives based on Bayesian concepts. Given their intuitive inferential basis and output,^6^, ^7^ these would seem obvious candidates to supplant the prevailing frequentist methodology. However, it is well‐known that the adoption of Bayesian methods continues to be hampered by several factors, such as the belief that advanced computational tools are required to make Bayesian statistics practical.^8^ The most persistent of these is that the full benefit of Bayesian methods demands specification of a prior level of belief, even in the absence of any appropriate insight. This “Problem of Priors” has cast a shadow over Bayesian methods since their emergence over 250 years ago,^9^ and has led to a variety of approaches, such as prior elicitation, prior sensitivity analysis, and objective Bayesian methodology; all have their supporters and critics.

One of the least well‐known was suggested over 70 years ago^10^ by one of the best‐known proponents of Bayesian methods during the 20th century, I. J. Good. It involves reversing the conventional direction of Bayes' theorem and determining the level of prior belief required to reach a specified level of posterior belief, given the evidence observed. This reversal of Bayes' theorem allows the assessment of new findings on the basis of whether the resulting prior is reasonable in the light of existing knowledge. Whether a prior is plausible in the light of existing knowledge can be assessed informally or more formally using techniques for comparing priors with existing data as suggested by Box^11^ and further refined by Evans and Moshonov,^12^ see also Nott et al.^13^, ^14^ for related approaches. Good stressed that despite the routine use of the adjectives “prior” and “posterior” in applications of Bayes' theorem, the validity of any resulting inference does not require a specific temporal ordering, as the theorem is simply a constraint ensuring consistency with the axioms of probability. While reversing Bayes' theorem is still regarded as unacceptable by some on the grounds it allows “cheating” in the sense of choosing priors to achieve a desired posterior inference,^15^ ^(p143)^ others point out this is not an ineluctable consequence of the reversal.^16^ ^(pp78−79)^ As we shall show, recent technical advances further weaken this criticism.

Good's belief in the value of Reverse‐Bayes methods won support from E.T. Jaynes in his well‐known treatise on probability. Explaining a specific manifestation of the approach (to be discussed shortly) Jaynes remarked: “We shall find it helpful in many cases where our prior information seems at first too vague to lead to any definite prior probabilities; it stimulates our thinking and tells us how to assign them after all”.^17^ ^(p126)^ Yet despite the advocacy of two leading figures in the foundations of Bayesian methodology, the potential of Reverse‐Bayes methods has remained largely unexplored. Most published work has focused on their use in putting new research claims in context, with Reverse‐Bayes methods being used to assess whether the prior evidence needed to make a claim credible is consistent with existing insight.^18^, ^19^, ^20^, ^21^, ^22^, ^23^, ^24^, ^25^, ^26^, ^27^, ^28^, ^29^, ^30^

The purpose of this paper is to highlight recent technical developments of Good's basic idea which lead to inferential tools of practical value in the analysis of summary measures as reported in meta‐analysis. As a running example we consider a recently published meta‐analysis investigating the association between corticosteroids and mortality in hospitalised patients with COVID‐19. Specifically, we show how Reverse‐Bayes methods address the current concerns about the interpretation of new findings and their replication. We begin by illustrating the basics of the Reverse‐Bayes approach for both hypothesis testing and parameter estimation. This is followed by a discussion of Reverse‐Bayes methods for assessing effect estimates in Section 2. These allow the credibility of both new and existing research findings reported in terms of NHST to be evaluated in the context of existing knowledge. This enables researchers to go beyond the standard dichotomy of statistical significance/non‐significance, extracting further insight from their findings. We then discuss the use of the Reverse‐Bayes approach in the most recalcitrant form of the Problem of Priors, involving the assessment of research findings which are unprecedented and thus lacking any clear source of prior support. We show how the concept of intrinsic credibility resolves this challenge, and puts recent calls to tighten *p*‐value thresholds on a principled basis.^31^ In Section 3 we describe Reverse‐Bayes methods with Bayes factors, the principled solution for Bayesian hypothesis testing. Finally, we describe in Section 4 Reverse‐Bayes approaches to interpretational issues that arise in conventional statistical analysis based on *p‐*values, and how they can be used to flag the risk of inferential fallacies. We close with some extensions and final conclusions.

### 1.1 Reverse‐Bayes for hypothesis testing

The subjectivity involved in the specification of prior distributions is often seen as a weak point of Bayesian inference. The Reverse‐Bayes approach can help to resolve this issue both in hypothesis testing and parameter estimation, we will start with the former.

Consider a null hypothesis $H_{0}$ with prior probability $\pi =\mathrm{Pr}\left(H_{0}\right)$, so $\mathrm{Pr}\left(H_{1}\right)=1-\pi$ is the prior probability of the alternative hypothesis $H_{1}.$ Computation of the posterior probability of $H_{1}$ is routine with Bayes' theorem:

$$\mathrm{Pr}\left(H_{1}|\;\text{data}\;\right)=\frac{\mathrm{Pr}\left(\;\text{data}\;|\;H_{1}\right)\mathrm{Pr}\left(H_{1}\right)}{\mathrm{Pr}\left(\;\text{data}|\;H_{0}\right)\mathrm{Pr}\left(H_{0}\right)+\mathrm{Pr}\left(\;\text{data}\;|\;H_{1}\right)\mathrm{Pr}\left(H_{1}\right)}.$$

Bayes' theorem can be written in more compact form as,

(1) $$\frac{\mathrm{Pr}\left(H_{1}\;|\;\text{data}\;\right)}{\mathrm{Pr}\left(H_{0}\;|\;\text{data}\;\right)}=\frac{\mathrm{Pr}\left(\;\text{data}\;|\;H_{1}\right)}{\mathrm{Pr}\left(\;\text{data}\;|\;H_{0}\right)}\;\frac{\mathrm{Pr}\left(H_{1}\right)}{\mathrm{Pr}\left(H_{0}\right)},$$

that is, the posterior odds are the likelihood ratio times the prior odds. The standard “forward‐Bayes” approach thus fixes the prior odds (or one of the underlying probabilities), determines the likelihood ratio for the available data, and takes the product to compute the posterior odds. Of course, the latter can be easily back‐transformed to the posterior probability $\mathrm{Pr}\left(H_{1}\;|\;\text{data}\;\right),$ if required. The Problem of Priors is now apparent: in order for us to update the odds in favour of $H_{1},$ we must first specify the prior odds. This can be problematic in situations where, for example, the evidence on which to base the prior odds is controversial or even non‐existent.

However, as Good emphasised it is entirely justifiable to “flip” Bayes' theorem around, allowing us to ask the question: Which prior, when combined with the data, leads to our specified posterior?

(2) $$\;\frac{\mathrm{Pr}\left(H_{1}\right)}{\mathrm{Pr}\left(H_{0}\right)}=\frac{\mathrm{Pr}\left(H_{1}|\;\text{data}\;\right)}{\mathrm{Pr}\left(H_{0}\;|\;\text{data}\;\right)}/\frac{\mathrm{Pr}\left(\;\text{data}|\;H_{1}\right)}{\mathrm{Pr}\left(\;\text{data}|\;H_{0}\right)}.$$

For illustration we re‐visit an example put forward by Good,^10^ ^(p35)^ perhaps the first published Reverse‐Bayes calculation. It centres on a question for which the setting of an initial prior is especially problematic: does an experiment provide convincing evidence for the existence of extra‐sensory perception (ESP)? The substantive hypothesis $H_{1}$ is that ESP exists, so that $H_{0}$ asserts it does not exist. Imagine an experiment in which a person has to make n consecutive guesses of random digits (between 0 and 9) and all are correct, as the ESP hypothesis $H_{1}$ would predict. The likelihood ratio is therefore,

(3) $$\frac{\mathrm{Pr}\left(\;\text{data}|\;H_{1}\right)}{\mathrm{Pr}\left(\;\text{data}\;|H_{0}\right)}=\frac{1}{{\left(1/10\right)}^{n}}=10^{n}.$$

It is unlikely that sceptics and advocates of the existence of ESP would ever agree on what constitutes reasonable priors from which to start a standard Bayesian analysis of the evidence. However, Good argued that Reverse‐Bayes offers a way forward by using it to set bounds on the prior probabilities for $H_{1}$ and $H_{0}.$ This is achieved via the outcome of a thought (Gedanken) experiment capable of demonstrating $H_{1}$ is more likely than $H_{0},$ that is, of leading to posterior probabilities such that $\mathrm{Pr}\left(H_{1}\;|\text{data}\;\right)>\mathrm{Pr}\left(H_{0}\;|\text{data}\;\right).$ Using this approach, which Good termed the *device of imaginary results*, we see that if the ESP experiment produced n=20 correct consecutive guesses, (2) combined with (3) implies that ESP may be deemed more likely than not to exist by anyone whose priors satisfy $\mathrm{Pr}\left(H_{1}\right)/\mathrm{Pr}\left(H_{0}\right)>10^{-20}.$ In contrast, if only n=3 correct guesses emerged, then the existence of ESP could be rejected by anyone whose priors satisfy $\mathrm{Pr}\left(H_{1}\right)/\mathrm{Pr}\left(H_{0}\right)<10^{-3}.$ Using Bayes' theorem in reverse has thus led to a quantitative statement of the prior beliefs that either advocates or sceptics of ESP must be able to justify in the face of results from a real experiment. The practical value of Good's approach was noted by Jaynes in his treatise: “[I]n the present state of development of probability theory, the device of imaginary results is usable and useful in a very wide variety of situations, where we might not at first think it applicable.”^17^ ^(p125−126)^

It is straightforward to extend (1) and (2) to hypotheses that involve unknown parameters θ. The likelihood ratio $\mathrm{Pr}\left(\;\text{data}|\;H_{1}\right)/\mathrm{Pr}\left(\;\text{data}|\;H_{0}\right)$ is then called a Bayes factor^32^, ^33^ where,

$$\mathrm{Pr}\left(\;\text{data}\;|H_{i}\right)=\int \mathrm{Pr}\left(\;\text{data}\;|\theta ,H_{i}\right)f\left(\theta |H_{i}\right)\mathrm{d\theta}$$

is the marginal likelihood under hypothesis $H_{i},$ i=0,1, obtained be integration of the ordinary likelihood with respect to the prior distribution $f\left(\theta |H_{i}\right).$ We will apply the Reverse‐Bayes approach to Bayes factors in Sections 3 and 4.

### 1.2 Reverse‐Bayes for parameter estimation

We can also apply the Reverse‐Bayes idea to continuous prior and posterior distributions of a parameter of interest θ. Reversing Bayes' theorem,

$$f\left(\theta \;|\text{data}\;\right)=\frac{f\left(\;\text{data}|\;\theta \right)f\left(\theta \right)}{f\left(\;\text{data}\;\right)}$$

then leads to

(4) $$f\left(\theta \right)=f\left(\;\text{data}\;\right)\;\frac{f\left(\theta \;|\text{data}\;\right)}{f\left(\;\text{data}\;|\theta \right)}.$$

So the prior is proportional to the posterior divided by the likelihood with proportionality constant $f\left(\;\text{data}\;\right).$

Consider Bayesian inference for the mean θ of a univariate normal distribution, assuming the variance $\sigma^{2}$ is known. Let x denote the observed value from that $N\left(\theta ,\sigma^{2}\right)$ distribution and suppose the prior for θ (and hence also the posterior) is conjugate normal. Each of them is determined by two parameters, usually the mean and the variance, but two distinct quantiles would also work. If we fix both parameters of the posterior, then the prior in (4) is—under a certain regularity condition—uniquely determined. For ease of presentation we work with the observational precision $\kappa =1/\sigma^{2}$ and denote the prior and posterior precision by δ and $\delta^{\prime },$ respectively. Finally let μ and $\mu^{\prime }$ denote the prior and posterior mean, respectively.

Forward‐Bayesian updating tells us how to compute the posterior precision and mean:

(5) $$\delta^{\prime }=\delta +\kappa ,$$

(6) $$\mu^{\prime }=\frac{\mu \delta +\mathrm{x\kappa}}{\delta^{\prime }}.$$

For example, fixed‐effect (FE) meta‐analysis is based on iteratively applying (5) and (6) to the summary effect estimate $x_{i}$ with standard error $\sigma_{i}$ from the *i*‐th study, i=1,…,n, starting with an initial precision of zero. Reverse‐Bayes simply inverts these equations, which leads to the following:

(7) $$\delta =\delta^{\prime }-\kappa ,$$

(8) $$\mu =\frac{\mu^{\prime }\delta^{\prime }-\mathrm{x\kappa}}{\delta },$$

provided $\delta^{\prime }>\kappa ,$ that is, the posterior precision must be larger than the observational precision.

**FIGURE 1:**
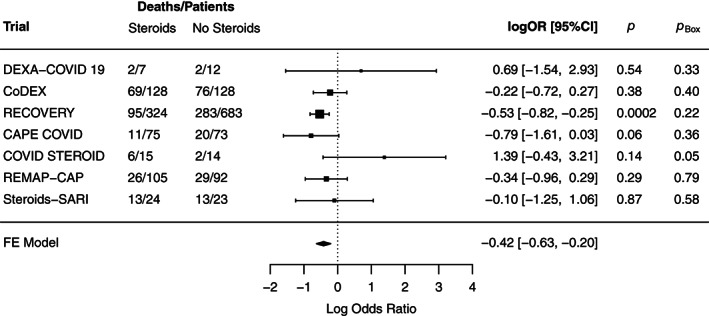
Forest plot of fixed‐effect meta‐analysis of n=7 randomised controlled trials investigating association between corticosteroids and mortality in hospitalised patients with COVID‐19.^34^ Shown are number of deaths among total number of patients for treatment/control group, log‐odds ratio effect estimates with 95% confidence interval, two‐sided *p‐*values *p*, and prior‐predictive tail probabilities $p_{\mathrm{Box}}$ with a meta‐analytic estimate based on the remaining studies serving as the prior

We will illustrate the application of (7) and (8) as well as the methodology in the rest of this paper using a recent meta‐analysis combining information from n=7 randomised controlled trials (RCTs) investigating the association between corticosteroids and mortality in hospitalised patients with COVID‐19^34^; its results are reproduced in Figure 1 (here and henceforth, odds ratios [ORs] are expressed as log odds ratios to transform the range from $\left(0,\infty \right)$ to $\left(-\infty ,+\infty \right),$ consistent with the assumption of normality). Let $x_{i}={\hat{\theta }}_{i}$ denote the maximum likelihood estimate (MLE) of the log odds ratio θ in the *i*‐th study with standard error $\sigma_{i}.$ The meta‐analytic odds ratio estimate under the fixed‐effect model (the pre‐specified primary analysis) is $\hat{\text{OR}}=0.66$ [95% CI: 0.53 to 0.82], respectively $\hat{\theta }=-0.42$ [95% CI: −0.63 to −0.20] for the log‐odds ratio θ, indicating evidence for lower mortality of patients treated with corticosteroids compared to patients receiving usual care or placebo. The pooled effect estimate $\hat{\theta }$ represents a posterior mean $\mu^{\prime }$ with posterior precision $\delta^{\prime }=83.8.$

With a meta‐analysis such as this, it is of interest to quantify potential conflict among the effect estimates from the different studies. To do this, we follow Presanis^35^ and compute a prior‐predictive tail probability^11^, ^12^ for each study‐specific estimate ${\hat{\theta }}_{i},$ with a meta‐analytic estimate based on the remaining studies serving as the prior. As discussed above, fixed‐effect meta‐analysis is standard forward‐Bayesian updating for normally distributed effect estimates with an initial flat prior. Hence, instead of fitting a reduced meta‐analysis for each study, we can simply use the Reverse‐Bayes Equations (7) and (8) together with the overall estimate to compute the parameters of the prior in the absence of the *i*‐th study (denoted by the index −i):


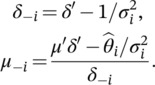




For example, through omitting the RECOVERY^36^ trial result ${\hat{\theta }}_{i}=-0.53$ with standard error $\sigma_{i}=0.145$ we obtain $\delta_{-i}=36.1$ and $\mu_{-i}=-0.26$. A prior‐predictive tail probability using the approach from Box^11^ is then obtained by computing $p_{\mathrm{Box}}=\mathrm{Pr}\left(\chi_{1}^{2}\geq t_{\mathrm{Box}}^{2}\right)$ with,

$$t_{\mathrm{Box}}=\frac{{\hat{\theta }}_{i}-\mu_{-i}}{\sqrt{\sigma_{i}^{2}+1/\delta_{-i}}}=-1.24.$$

This leads to $p_{\mathrm{Box}}=0.22$ for the RECOVERY trial, indicating very little prior‐data conflict. The tail probabilities for the other studies are even larger, with the exception of the COVID STEROID trial $\left(p_{\mathrm{Box}}=0.05\right),$ see Figure 1. The lack of strong conflict can be seen as an informal justification of the assumptions of the underlying fixed‐effect meta‐analysis.^35^, ^37^ A related method in network meta‐analysis is to assess consistency via “node‐splitting.”^38^ Reverse‐Bayes methods may also be useful for conflict assessment in more general evidence synthesis methods where multiple distinct sources of data are combined,^39^, ^40^ but this may require more advanced numerical techniques.

Instead of determining the prior completely based on the posterior, one may also want to fix one parameter of the posterior and one parameter of the prior. This is of particular interest in order to challenge “significant” or “non‐significant” findings through the Analysis of Credibility, as we will see in the following section.

## 2 REVERSE‐BAYES METHODS FOR THE ASSESSMENT OF EFFECT ESTIMATES

A more general question amenable to Reverse‐Bayes methods is the assessment of effect estimates and their statistical significance or non‐significance. This issue has recently attracted intense interest following the public statement of the American Statistical Association about the misuse and misinterpretation of the NHST concepts of statistical significance and non‐significance.^3^ First investigated 20 years ago by Matthews,^19^, ^20^ Reverse‐Bayes methods for assessing both statistically significant and non‐significant findings have been termed the Analysis of Credibility^41^ (or AnCred), whose principles and practice we now briefly review.

### 2.1 The analysis of credibility

Suppose the study gives rise to a conventional confidence interval for the unknown effect size θ at level 1−α with lower limit L and upper limit U. Assume that L and U are symmetric around the point estimate $\hat{\theta }$ (assumed to be normally distributed with standard error σ). AnCred then takes this likelihood and uses a Reverse‐Bayes approach to deduce the prior required in order to generate credible evidence for the existence of an effect, in the form of a posterior that excludes no effect. As such, AnCred allows evidence deemed *statistically significant*/*non‐significant* in the NHST framework to be assessed for its *credibility* in the Bayesian framework. As the latter conditions on the data rather than the null hypothesis, it is inferentially directly relevant to researchers. After a suitable transformation AnCred can be applied to a large number of commonly used effect measures such as differences in means, odds ratios, relative risks and correlations. We refer to the literature of meta‐analysis for details about conversion among effect size scales, for example, Cooper et al.^42^ ^(ch11.6)^ The inversion of Bayes' theorem needed to assess credibility requires the form and location of the prior distribution to be specified. This in turn depends on whether the claim being assessed is statistically significant or non‐significant; we consider each below.

#### 2.1.1 Challenging statistically significant findings

A statistically significant finding at level α is characterised by both L and U being either positive or negative. Equivalently $z^{2}>z_{\alpha /2}^{2}$ is required where $z=\hat{\theta }/\sigma$ denotes the corresponding test statistic and $z_{\alpha /2}$ the $\left(1-\alpha /2\right)‐\text{quantile}$ of the standard normal distribution.

For significant findings, the idea is to ask how sceptical we would have to be not to find the apparent effect estimate convincing. To this end, a *sceptical prior* is derived such that the corresponding posterior credible interval just includes zero, the value of no effect. This critical prior interval can then be compared with internal or external evidence to assess if the finding is credible or not, despite being “statistically significant.”

More specifically, a Reverse‐Bayes approach is applied to significant confidence intervals (at level *α*) based on a normally distributed effect estimate. The sceptical prior is a mean‐zero normal distribution with variance $\tau^{2}=g\cdot \sigma^{2},$ so the only free parameter is the relative prior variance $g=\tau^{2}/\sigma^{2}.$ The posterior is hence also normal and either its lower α/2‐quantile (for positive $\hat{\theta })$ or upper 1−α/2‐quantile (for negative $\hat{\theta })$ is fixed to zero, so just represents “non‐credible.” The sufficiently sceptical prior then has relative variance,

(9) $$g=\left\{\begin{array}{cc} \frac{1}{z^{2}/z_{\alpha /2}^{2}-1} & \;\text{if}\;z^{2}>z_{\alpha /2}^{2}\\ \text{undefined} & \text{else} \end{array}\right.$$

see the Appendix in Held^27^ for a derivation. The corresponding *scepticism limit*,^41^ the upper bound of the equal‐tailed sceptical prior credible interval at level 1−α, is,

(10) $$\;\mathrm{SL}=\frac{{\left(U-L\right)}^{2}}{4\sqrt{\mathrm{UL}}},$$

which holds for any value of α provided the effect is significant at that level.

**FIGURE 2:**
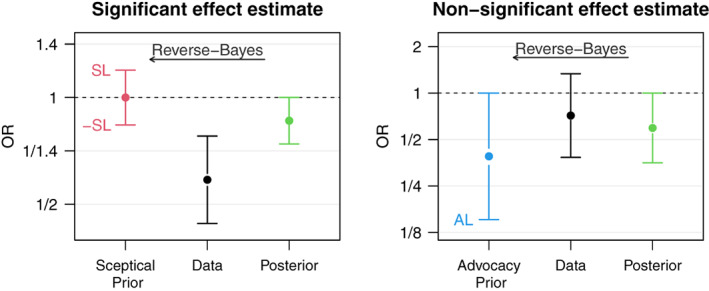
Two examples of the Analysis of Credibility. Shown are point estimates within 95% confidence/credible intervals. The left plot illustrates how a sceptical prior is used to challenge the significant finding from the RECOVERY trial.^36^ The right plot illustrates how an advocacy prior is used to challenge a non‐significant finding from the REMAP‐CAP trial.^43^ In both scenarios the posterior is fixed to be just non‐credible/credible [Colour figure can be viewed at wileyonlinelibrary.com]

The left plot in Figure 2 illustrates the AnCred procedure for the finding from the RECOVERY trial,^36^ the only statistically significant result (at the conventional α=0.05 level) shown in Figure 1. The trial found a decrease in COVID‐19 mortality for patients treated with corticosteroids compared to usual care or placebo ($\hat{\theta }=-0.53$ [95% CI: −0.82 to −025]). The sufficiently sceptical prior has relative variance g=0.39, so the sufficiently sceptical prior variance needs to be roughly 2.5 times smaller than the variance of the estimate to make the result non‐credible. The scepticism limit on the log‐odds ratio scale turns out to be SL=0.18, which corresponds to a critical prior interval with limits 0.84 and 1.19 on the odds ratio scale. Thus sceptics may still reject the RECOVERY trial finding as lacking credibility despite its statistical significance if external evidence suggests mortality reductions (in terms of odds) are unlikely to exceed 1−0.84≈16%.

It is also possible to apply the approach to the meta‐analytic log‐odds ratio estimate $\hat{\theta }=-0.42$ (95% CI: −0.63 to −0.20) from all seven studies combined. Then SL=0.13, so the meta‐analytic estimate can be considered as non‐credible if external evidence suggests that mortality reductions are unlikely to exceed $1-\exp\left(-\mathrm{SL}\;\right)=1-0.88\approx 12\%.$ This illustrates that the meta‐analytic estimate has gained credibility compared to the result from the RECOVERY study alone, despite the reduction in the effect estimate $(\hat{\text{OR}}=\exp(\hat{\theta })=0.66$ vs. 0.59 in the RECOVERY study).

#### 2.1.2 Challenging statistically non‐significant findings

It is also possible to challenge “non‐significant” findings (i.e., those for which the CI now includes zero, so $z^{2}<z_{\alpha /2}^{2}$) using a prior that pushes the posterior toward being credible in the Bayesian sense, with posterior credible interval no longer including zero, corresponding to no effect.

Matthews^41^ proposed the “advocacy prior” for this purpose, a normal prior with positive mean μ and variance $\tau^{2}$ chosen such that the α/2‐quantile is fixed to zero (for positive effect estimates $\hat{\theta }>0$). He showed that the “advocacy limit” AL, the $\left(1-\alpha /2\right)‐\text{quantile}$ of the advocacy prior is,

(11) $$\;\mathrm{AL}=-\frac{U+L}{2\;\mathrm{UL}}{\left(U-L\right)}^{2}$$

to reach credibility of the corresponding posterior at level α. We show in Appendix A.1 that the corresponding relative prior mean $f=\mu /\hat{\theta }$ is,

(12) $$f=\left\{\begin{array}{cc} \frac{2}{1-z^{2}/z_{\alpha /2}^{2}} & \;\text{if}\;z^{2}<z_{\alpha /2}^{2}\\ \text{undefined} & \text{else}. \end{array}\right.$$

There are two important properties of the advocacy prior. First, the coefficient of variation CV is,

$$\;\mathrm{CV}=\tau /\mu =z_{\alpha /2}^{-1}.$$

The advocacy prior $\theta ∼N\left(\mu ,\tau^{2}=\mu^{2}\;{\mathrm{CV}}^{2}\right)$ is hence characterised by a fixed coefficient of variation, so this prior has equal evidential weight (quantified in terms of $\mu /\tau =z_{\alpha /2})$ as data which are “just significant” at level α. Second, the AL defines the family of normal priors capable of rendering a “non‐significant” finding credible at the same level. Such priors are summarised by the credible interval $\left(L_{o},U_{o}\right)$ where $L_{o}\geq 0,$ $U_{o}\leq \mathrm{AL}.$ Thus when confronted with a “non‐significant” result—often, and wrongly, interpreted as indicating no effect—advocates of the existence of an effect may still claim the existence of the effect is credible to the same level if there exists prior evidence or insight compatible with the credible interval $\left(L_{o},U_{o}\right).$ If the evidence for an effect is strong (weak), the resulting advocacy prior will be broad (narrow), giving advocates of an effect more (less) latitude to make their case under terms of AnCred. Note that (11) and (12) also hold for negative effect estimates, where we fix the $\left(1-\alpha /2\right)‐\text{quantile}$ of the advocacy prior to zero and define the AL as the α/2‐quantile of the advocacy prior.

For illustration we consider the data from the REMAP‐CAP trial^43^ that supported the RECOVERY trial finding of decreased COVID‐19 mortality from corticosteroid use. However, this trial involved far fewer patients, and despite the point estimate showing efficacy, the relatively large uncertainty rendered the overall finding non‐significant at the 5% level ($\hat{\theta }=-0.34$ [95% CI: −0.96 to 0.29]). Such an outcome is frequently (and wrongly) taken to imply no effect. The use of AnCred leads to a more nuanced conclusion. The AL on the log‐odds ratio scale for REMAP‐CAP is −1.89, that is, 0.15 on the odds ratio scale, see also the right plot in Figure 2. Thus advocates of the effectiveness of corticosteroids can regard the trial as providing credible evidence of effectiveness despite its non‐significance if external evidence supports mortality reductions (in terms of odds) in the range 0% to 85%. So broad an advocacy range reflects the fact that this relatively small trial provides only modest evidential weight, and thus little constraint on prior beliefs about the effectiveness of corticosteroids.

Another way to push non‐significant findings toward credibility is to use a prior based on data from another study or a different subgroup. For example, Best et al.^30^ consider results from the MENSA trial^44^ on the efficacy of Mepolizumab against placebo in 551 adult and 25 adolescent patients with severe asthma. The treatment effect was estimated to be positive in both subgroups but lacked significance among adolescents. Best et al. combine the data in the adolescent subgroup with a mixture prior based on a weak and an informative component. The weak component is a minimally informative normal prior with mean zero and large variance. The variance is chosen such that the information content of the prior is equivalent to that provided by a single subject or event (*unit‐information prior*).^45^ The other component is an informative prior based on the (significant) results from the adolescent subgroup. A Reverse‐Bayes approach is used to determine how much prior weight one needs to assign to the informative component to obtain a credible posterior result with a 95% highest posterior credible interval no longer including zero. In the MENSA trial the required prior weight on the informative component was 0.7 (and thus 0.3 on the weak prior component) to achieve a credible result.^30^ This illustrates that a considerable amount of “Bayesian borrowing” is required to extrapolate the results from adults to adolescents.

In the meta‐analytic setting we may ask a similar question: Suppose we want to combine the REMAP‐CAP study results with a fraction of the RECOVERY trial data, how much weight do we need to assign to the RECOVERY trial to make the REMAP‐CAP study credible? The unit‐information prior for a logOR has variance $\tau^{2}=4$ (see Section  2.4.1 in Spiegelhalter et al.^21^), so the mixture prior is,

$$\theta ∼w\cdot N\left(0,\tau^{2}=4\right)+\left(1-w\right)\cdot N\left({\hat{\theta }}_{\mathrm{REC}},\sigma_{\mathrm{REC}}^{2}\right)$$

with w the mixing weight and point estimate ${\hat{\theta }}_{\mathrm{REC}}$ and squared standard error $\sigma_{\mathrm{REC}}^{2}$ of the RECOVERY trial, respectively. The resulting posterior is again a mixture of two normals with the posterior mean and variance of each component being the usual ones obtained from conjugate Bayesian updating, while the weights are proportional to the marginal likelihood of the data under each component (see Section  3.5 in Best et al.^30^ for details).

**FIGURE 3:**
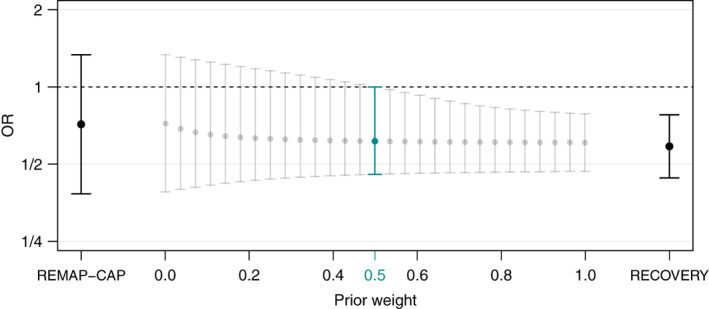
Illustration of the Reverse‐Bayes borrowing method. The data from the (non‐significant) REMAP‐CAP trial are combined with a mixture prior consisting of the (significant) RECOVERY trial data and a unit‐information prior (both estimates shown with 95% confidence interval). The resulting posterior medians with equal‐tailed 95% credible intervals are shown for a range of mixing weights. The Reverse‐Bayes mixing weight w=0.5 leads to the highlighted posterior with upper credible interval limit fixed at one [Colour figure can be viewed at wileyonlinelibrary.com]

Figure 3 shows posterior medians with 95% equal‐tailed credible intervals for a range of mixing weights. We see that a weight of at least w=0.5 is required to render the resulting posterior credible. Advocates of corticosteroids thus need to be able to justify such levels of prior beliefs, in order to conclude efficacy of corticosteroids also in the REMAP‐CAP trial.

#### 2.1.3 Assessing credibility via equivalent prior study sizes

Reverse‐Bayes credibility assessments can also be formulated in terms of the size and content of a prior study capable of challenging a claim of statistical significance/non‐significance. This approach puts the weight of prior evidence in the context of the observed data, expressed as participant numbers. Greenland^22^ demonstrated the value of this approach in assessing the credibility of statistically significant findings from large observational studies in epidemiology. The same concept can, however, be extended to the assessment of both significant and non‐significant outcomes more widely, such as small RCTs. For any study generating binary data in the form of event/non‐event counts under two different conditions, the comparative effect measure can be expressed as a log‐odds ratio with variance (squared standard error),

(13) $$1/m_{1}+1/n_{1}+1/m_{2}+1/n_{2}.$$

where $m_{i}$ and $n_{i}$ are the numbers of events and non‐events, respectively, in study arm i=1,2. This provides the link between the Reverse‐Bayes prior distribution and the corresponding numbers of prior study participants. Using the simplifying assumptions of equal numbers of events $m=m_{1}=m_{2}$ and large numbers of non‐events $n_{1}$ and $n_{2}$ in each arm $(n_{i}>100\;m_{i},$ say), the variance (13) reduces to 2/m. The Reverse‐Bayes sceptical prior defined in Section 2.1.1 has variance $\tau^{2}={\mathrm{SL}}^{2}/z_{\alpha /2}^{2},$ where SL is the sceptical limit. Equating the two implies that such a prior is equivalent to a (large) hypothetical study with,

$$m=2/\tau^{2}=2\;z_{\alpha /2}^{2}/{\mathrm{SL}}^{2}$$

events in both arms. The more compelling the data—that is, the smaller the value of SL—the larger the number of events m required in both arms of the hypothetical large prior study to render the result non‐credible at level α.

While the assumption of large studies can be appropriate with epidemiological studies involving rare events, it can be harder to justify for RCTs. Fortunately, the theory can be extended to encompass these and also the case of non‐significant findings with little additional complexity. In the case of the sceptical prior, we simply require that the numbers of events m and non‐events n are the same in both arms to constrain the mean to zero; the variance (13) then simplifies to 2/m+2/n. Adding the constraint that the event rate of the sceptical prior $R=m/\left(m+n\right)$ matches that of the study under assessment, we then find,

$$m=\frac{2}{\tau^{2}\left(1-R\right)}\;\text{and}\;n=\frac{m\left(1-R\right)}{R}.$$

For example, from Figure 1 the RECOVERY trial has an overall mortality rate $R=\left(95+283\right)/\left(324+683\right)=37.54\%$ and SL=0.178 at the α=5% level $\left(z_{\alpha /2}=1.96\right)$ corresponds to $\tau^{2}=0.091^{2},$ so m=389 and n=648 (these are integer approximations of exact computations), and thus a prior study capable of challenging the credibility of the RECOVERY trial requires 1037 patients and 389 deaths in each arm. At more than twice the size of the RECOVERY trial (2074 vs. 1007) patients and considerably more deaths in both arms, this level of sceptical prior evidence highlights the robustness of the trial finding.

A similar approach determines the characteristics of the hypothetical prior study needed to turn a “negative” non‐significant finding into one that is credible to a specific α level. The Reverse‐Bayes advocacy prior from Matthews^41^ described in Section 2.1.2 has a mean μ=AL/2 and variance $\tau^{2}={\mathrm{AL}}^{2}/{\left(2z_{\alpha /2}\right)}^{2}.$ Under the large study assumption and equating the latter with (13) as before, the corresponding number of events needed to be observed in both arms of the hypothetical study is $m=2/\tau^{2}=8\;z_{\alpha /2}^{2}/{\mathrm{AL}}^{2}.$ To incorporate the non‐zero mean by which this prior represents advocacy, these m events are taken to have been observed among participants allocated to the two study arms in the ratio 1:K where $K=\exp\left(\mu \right)=\exp\left(\;\mathrm{AL}/2\right),$ the allocation being such that it increases the relative evidential weight for the hypothesis “negated” by the non‐significance.

As before, while the large study approximation may be justified in epidemiological examples, this is less likely to be true for RCTs. In such cases, we can adapt the approach used for sceptical priors, the size and composition of the advocacy prior being found by setting the numbers of events m in each arm the same, but this time allowing for different numbers of non‐events in each arm via the allocation ratio K. The resulting variance is then.

$$\tau^{2}=\frac{2}{m}+\frac{K+1}{n}$$

where n is the number of non‐events in the arm used to support the null hypothesis (e.g., the control arm in an RCT). With the control arm event rate $R=m/\left(m+n\right)$ constrained to match that of the actual study, we find,

$$m=\frac{2-R\left(1-K\right)}{\tau^{2}\left(1-R\right)}\;\text{and}\;n=\frac{m\left(1-R\right)}{R}.$$

As an example, we return to the REMAP‐CAP trial, whose findings were consistent with a reduction of mortality but failed to reach statistical significance. As noted above, its advocacy limit (AL = −1.89) implies this trial has relatively little evidential weight, and gives considerable scope for prior studies to make its outcome credible at the 95% level. With $K=\exp\left(\mu \right)=\exp\left(\;\mathrm{AL}/2\right)=0.39,$ R=29/92 and $z_{\alpha /2}=1.96$ we find m=11 and n=25. Thus the hypothetical prior study comprises 11 deaths from 11 + 25 = 36 patients in the control arm and the same number of deaths from $11+\left(25/0.4\right)=75$ patients in the treatment arm. At barely half the total size of REMAP‐CAP but a considerably more impressive mortality reduction from R=29/92=32% in the control arm to 11/75=15% in the treatment arm (rather than 26/105=25% in REMAP‐CAP), the nature of this hypothetical prior study confirms the paucity of evidence in the original trial.

#### 2.1.4 The fail‐safe *N* method

Another data representation of a sceptical prior forms the basis of the well‐known “fail‐safe *N*” method, sometimes also called “file‐drawer analysis.” This method, first introduced by Rosenthal^46^ and later refined by Rosenberg,^47^ is commonly applied to the results from a meta‐analysis and answers the question: “How many unpublished negative studies do we need to make the meta‐analytic effect estimate non‐significant?” A relatively large *N* of such unpublished studies suggests that the estimate is robust to potential null‐findings, for example due to publication bias. Calculations are made under the assumption that the unpublished studies have an average effect of zero and a precision equal to the average precision of the published ones.

While the method does not identify nor adjust for publication bias, it provides a quick way to assess how robust the meta‐analytic effect estimate is. The method is available in common software packages such as metafor ^48^ and its simplicity and intuitive appeal have made it very popular among researchers.

AnCred and the fail‐safe *N* are both based on the idea to challenge effect estimates such that they become “non‐significant/not credible,” and it is easy to show that the methods are under some circumstances also technically equivalent. To illustrate this, we consider again the meta‐analysis on the association between corticosteroids and COVID‐19 mortality^34^ which gave the pooled log‐odds ratio estimate $\hat{\theta }=-0.42$ with standard error σ=0.11, posterior precision $\delta^{\prime }=83.8$ and test statistic $z=\hat{\theta }/\sigma =-3.81.$

Using the Rosenberg^47^ approach (as implemented in the fsn() function from the metafor package) we find that at least N=20 additional but unpublished non‐significant findings are needed to make the published meta‐analysis effect non‐significant. If instead, we challenge the overall estimate with AnCred, we obtain the relative prior variance g=0.36 using Equation (9), so $\tau^{2}=0.0043.$ Taking into account the average precision $\delta^{\prime }/n=11.98$ of the different effect estimates estimates in the meta‐analysis leads to $N=n/\left(\delta^{\prime }\cdot \tau^{2}\right)=19.5$ which is equivalent to the fail‐safe N result after rounding to the next larger integer.

### 2.2 Intrinsic credibility

The Problem of Priors is at its most challenging in the context of entirely novel “out of the blue” effects for which no obviously relevant external evidence exist. By their nature, such findings often attract considerable interest both within and beyond the research community, making their reliability of particular importance. Given the absence of external sources of evidence, Matthews^41^ proposed the concept of *intrinsic credibility*. This requires that the evidential weight of an unprecedented finding is sufficient to put it in conflict with the sceptical prior rendering it non‐credible. In the AnCred framework, this implies a finding possesses intrinsic credibility at level α if the estimate $\hat{\theta }$ is outside the corresponding sceptical prior interval $\left[-\mathrm{SL},\mathrm{SL}\;\right]$ extracted using Reverse‐Bayes from the finding itself, i. e. ${\hat{\theta }}^{2}>{\mathrm{SL}}^{2}$ with SL given in (10). Matthews showed this implies an unprecedented finding is intrinsically credible at level α=0.05 if its *p‐*value does not exceed 0.013.

Held^27^ refined the concept by suggesting the use of a prior‐predictive check^11^, ^12^ to assess potential prior‐data conflict. With this approach the uncertainty of the estimate $\hat{\theta }$ is also taken into account since it is based on the prior‐predictive distribution, in this case $\hat{\theta }∼N\left(0,\sigma^{2}+\tau^{2}=\sigma^{2}\;\left(1+g\right)\right)$ with g as given in (9). Intrinsic credibility is declared if the (two‐sided) tail probability,

$$p_{\mathrm{Box}}=\mathrm{Pr}\left(\chi_{1}^{2}\geq {\hat{\theta }}^{2}/\left(\sigma^{2}+\tau^{2}\right)\right)=\mathrm{Pr}\left(\chi_{1}^{2}\geq z^{2}/\left(1+g\right)\right)$$

of $\hat{\theta }$ under the prior‐predictive distribution is smaller than α. It turns out that the *p‐*value associated with θ needs to be at least as small as 0.0056 to obtain intrinsic credibility at level α=0.05, providing another principled argument for the recent proposition to lower the *p‐*value threshold for the claims of new discoveries to 0.005.^31^ A simple check for intrinsic credibility is based on the *credibility ratio*, the ratio of the upper to the lower limit (or vice versa) of a confidence interval for a significant effect size estimate. If the credibility ratio is smaller than 5.8 then the result is intrinsically credible.^27^ This holds for confidence intervals at all possible values of α, not just for the 0.05 standard. For example, in the RECOVERY study the 95% confidence interval for the log‐odds ratio ranges from −0.82 to −0.25, so the credibility ratio is −0.82/−0.25=3.27<5.8 and the result is intrinsically credible at the standard 5% level.

#### 2.2.1 Replication of effect direction

Whether intrinsic credibility is assessed based on the prior or the prior‐predictive distribution, it depends on the level α in both cases. To remove this dependence, Held^27^ proposed to consider the smallest level at which intrinsic credibility can be established, defining the *p‐*value for intrinsic credibility,

(14) $$p_{\mathrm{IC}}=2\left\{1-\Phi \left(\frac{\left|z\right|}{\sqrt{2}}\right)\right\},$$

see Section 4 in Held^27^ for the derivation. Now $z=\hat{\theta }/\sigma$, so compared to the standard *p‐*value $p=2\left\{1-\Phi \left(\left|z\right|\right)\right\}$, the *p‐*value for intrinsic credibility is based on twice the variance $\sigma^{2}$ of the estimate $\hat{\theta }$. Although motivated from a different perspective, inference based on intrinsic credibility thus mimics the *doubling the variance rule* advocated by Copas and Eguchi^49^ as a simple means of adjusting for model uncertainty.

Moreover, Held^27^ showed that $p_{\mathrm{IC}}$ is connected to $p_{\mathrm{rep}}$ of Killeen,^50^ the probability that a replication will result in an effect estimate ${\hat{\theta }}_{r}$ in the same direction as the observed effect estimate $\hat{\theta }$, by $p_{\mathrm{rep}}=1-p_{\mathrm{IC}}/2.$ Hence, an intrinsically credible estimate at a small level α will have high chance of replicating since $p_{\mathrm{rep}}\geq 1-\alpha /2.$ Note that $p_{\mathrm{rep}}$ lies between 0.5 and 1 with the extreme case $p_{\mathrm{rep}}=0.5$ if $\hat{\theta }=0.$

As an example, the *p‐*value for intrinsic credibility for the RECOVERY trial finding (with *p‐*value p=0.0002) cited earlier is $p_{\mathrm{IC}}=0.01$ and thus the probability of the replication effect going in the same direction (i.e., reduced mortality in this case) is 0.995. In contrast, the finding from the smaller REMAP‐CAP trial (with p=0.29) leads to $p_{\mathrm{IC}}=0.46,$ and the probability of effect direction replication is hence only 0.77.

## 3 REVERSE‐BAYES METHODS WITH BAYES FACTORS

The AnCred procedure as described above uses posterior credible intervals as a means of quantifying evidence. However, quantification of evidence with Bayes factors is a more principled solution for hypothesis testing in the Bayesian framework.^32^, ^33^ Bayes factors enable direct probability statements about null and alternative hypothesis and they can also quantify evidence *for* the null hypothesis, both are impossible with indirect measures of evidence such as *p‐*values.^51^ Reverse‐Bayes approaches combined with Bayes factor methodology was pioneered by Carlin and Louis^18^ but then remained unexplored until Pawel and Held^29^ proposed an extension of AnCred where Bayes factors are used as a means of quantifying evidence. Rather than determining a prior such that a finding becomes “non‐credible” in terms of a posterior credible interval, this approach determines a prior such that the finding becomes “non‐compelling” in terms of a Bayes factor. In the second step of the procedure, the plausibility of this prior is quantified using external data from a replication study. Here, we will illustrate the methodology using only an original study; we mention extensions for replications in Section 5.1.

### 3.1 Sceptical priors

As before, $\hat{\theta }$ denotes the estimate of the unknown mean θ of a $N\left(\theta ,\sigma^{2}\right)$ distribution with known variance $\sigma^{2}.$ A standard hypothesis test compares the null hypothesis $H_{0}:\theta =0$ to the alternative $H_{1}:\theta \neq 0.$ Bayesian hypothesis testing requires specification of a prior distribution of θ under $H_{1}.$ A typical choice is a local alternative, a unimodal symmetric prior distribution centred around the null value.^52^ We consider again the conjugate sceptical prior $\theta |H_{1}∼N\left(0,\tau^{2}=g\cdot \sigma^{2}\right)$ with relative prior variance g for this purpose. This leads to the Bayes factor comparing $H_{0}$ to $H_{1}$ being,

(15) $${\mathrm{BF}}_{01}=\sqrt{1+g}\cdot \exp\left\{-\frac{g}{1+g}\cdot \frac{z^{2}}{2}\right\},$$

where $z=\hat{\theta }/\sigma .$ Yet again, the amount of evidence which the data provide against the null hypothesis depends on the prior parameter g; As g becomes smaller $\left(g↓0\right),$ the null hypothesis and the alternative will become indistinguishable, so the data are equally likely under both $\left({\mathrm{BF}}_{01}\to 1\right).$ On the other hand, for increasingly diffuse priors $\left(g\to \infty \right),$ the null hypothesis will always prevail $\left({\mathrm{BF}}_{01}\to \infty \right)$ due to the Jeffreys‐Lindley paradox.^53^ In between, the ${\mathrm{BF}}_{01}$ reaches a minimum at $g=\max\left\{z^{2}-1,0\right\}$ leading to,

(16) $${\text{minBF}}_{01}=\left\{\begin{array}{ll} \left|z\right|\cdot \exp\left\{-z^{2}/2\right\}\cdot \sqrt{e} & \text{if }\left|z\right|>1\\ 1 & \text{else} \end{array}\right.$$

which is an instance of a *minimum Bayes factor*, the smallest possible Bayes factor within a class of alternative hypotheses, in this case zero mean normal alternatives.^51^, ^54^, ^55^, ^56^

Reporting of minimum Bayes factors is one attempt of solving the Problem of Priors in Bayesian inference. However, this bound may be rather small and the corresponding prior unrealistic. In contrast, the Reverse‐Bayes approach makes the choice of the prior explicit by determining the relative prior variance parameter g such that the finding is no longer compelling, followed by assessing the plausibility of this prior. To do so, one first fixes ${\mathrm{BF}}_{01}=\gamma ,$ where γ is a cut‐off above which the result is no longer convincing, for example γ=1/10, the level for strong evidence according to the classification from Jeffreys.^32^ The sufficiently sceptical relative prior variance is then given by,

(17) $$\begin{array}{l} g=\left\{\begin{array}{ll} -\frac{z^{2}}{q}-1 & \;\text{if}-\frac{z^{2}}{q}\geq 1\\ \text{undefined} & \text{else} \end{array}\right.\\ \text{where}\;q=W\left(-\frac{z^{2}}{\gamma^{2}}\cdot \exp\left\{-z^{2}\right\}\right) \end{array}$$

where $W\left(\cdot \right)$ is the branch of the Lambert W function that satisfies $W\left(y\right)\leq -1$ for $y\in \left[-e^{-1},0\right),$ ^57^ see the Appendix in Pawel and Held^29^ for a proof.

The sufficiently sceptical relative prior variance g exists only for a cut‐off γ if ${\text{minBF}}_{01}\leq \gamma ,$ similar to standard AnCred where it exists only at level α if the original finding was significant at the same level. In contrast to standard AnCred, however, if the sufficiently sceptical relative prior variance g exists, there are always two solutions, a consequence of the Jeffreys‐Lindley paradox: If ${\mathrm{BF}}_{01}$ decreases in g below the chosen cut‐off γ, after attaining its minimum it will monotonically increase and intersect a second time with γ, admitting a second solution for the sufficiently sceptical prior.

**FIGURE 4:**
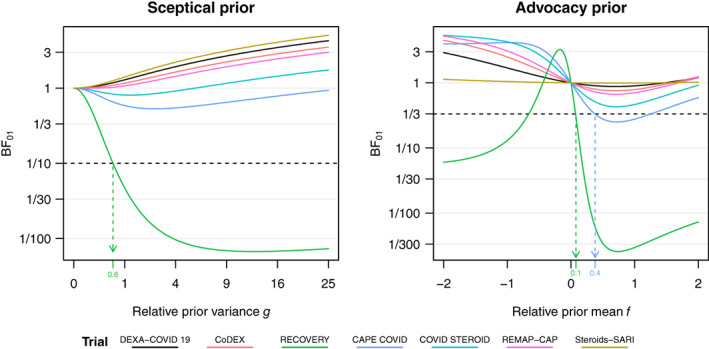
Illustration of the AnCred with Bayes factors procedure using the findings from the meta‐analysis on the association of COVID‐19 mortality and corticosteroids. The left plot shows the Bayes factor ${\mathrm{BF}}_{01}$ as a function of the relative variance $g=\tau^{2}/\sigma^{2}$ of the sceptical prior. The result from the RECOVERY trial is challenged with a sceptical prior such that ${\mathrm{BF}}_{01}=1/10,$ for the other trials such a prior does not exist. The right plot shows the Bayes factor ${}_{01}$ as a function of the relative mean $f=\mu /\hat{\theta }$ of the advocacy prior where the coefficient of variation from the prior is fixed to $\mathrm{CV}=\tau /\mu =1/z\left(\gamma =1/3\right)=0.67,$ where $z\left(\gamma \right)$ is given in (20). The RECOVERY and the CAPE COVID findings are challenged such that ${\mathrm{BF}}_{01}=1/3$, for the other trials such a prior does not exist [Colour figure can be viewed at wileyonlinelibrary.com]

We now re‐visit the meta‐analysis example considered earlier: The left plot in Figure 4 shows the Bayes factor ${\mathrm{BF}}_{01}$ from (15) as a function of the relative prior variance g for each finding included in the meta‐analysis. Most of them did not include a great number of participants and thus provide little evidence against the null hypothesis for any value of the relative prior variance g. In contrast, the finding from the RECOVERY trial^36^ provides more compelling evidence and can be challenged up to ${\text{minBF}}_{01}=1/148.9.$ For example, we see in Figure 4 that the relative sceptical prior variance needs to be g≤0.59 such that the finding is no longer compelling at level γ=1/10. This translates to a 95% prior credible interval from 0.8 to 1.24 for the OR (or any narrower interval around 1). Hence, a sceptic might still consider the RECOVERY finding to be unconvincing, despite its minimum BF being very compelling, if external evidence supports ORs in that range. By applying the prior‐to‐data conversion method described in Section 2.1.3 we can further see that the evidential value of this prior is equivalent to a trial with 258 events and 429 non‐events in both arms (so that the overall mortality rate is equivalent with the RECOVERY trial). For comparison, the sceptical prior from standard AnCred at α=0.05 was equivalent to a trial with 389 events and 648 non‐events, respectively.

The plausibility of the sufficiently sceptical prior can be evaluated in light of external evidence, but what should we do in the absence of such? We could again use the Box^11^ prior‐predictive check as in Section 2.2, however, the resulting tail probability is difficult to compare to the Bayes‐factor cut‐off γ. When a specific alternative model to the null is in mind, Box^11^ ^(p391)^ also suggested to use a Bayes factor for model criticism of the null model. Following this approach, Pawel and Held^29^ proposed to define a second Bayes factor contrasting the sufficiently sceptical prior to an optimistic prior, which they defined as $\theta |H_{2}∼N\left(\hat{\theta },\sigma^{2}\right)$ the posterior of θ based on the data and the reference prior $f\left(\theta \right)\propto 1$. The optimistic prior therefore represents the position of a proponent who takes the original claim at face value. This leads to the second Bayes factor being,

(18) $${\mathrm{BF}}_{12}=\sqrt{\frac{2}{1+g}}\cdot \exp\left\{-\frac{1}{2}\cdot \frac{z^{2}}{1+g}\right\}.$$

Analogously to the tail probability approach from Section 2.2, intrinsic credibility is established if the data support the optimistic over the sceptical prior at a higher level than they support the sceptical prior over the null hypothesis, that is, if,

$${\mathrm{BF}}_{12}\leq {\mathrm{BF}}_{01}$$

with sufficiently sceptical relative prior variance g from (17) used in both Bayes factors. For example, if we challenge the RECOVERY trial finding such that the resulting Bayes factor is only ${\mathrm{BF}}_{01}=1/10,$ we obtain with (9) the sufficiently sceptical relative prior variance g=0.59 and in turn with (18) the Bayes factor ${\mathrm{BF}}_{12}=1/64,$ so the finding is intrinsically credible at γ=1/10.

To remove the dependence on the choice of the level γ, one can determine the smallest level γ where intrinsic credibility can be established. This defines a Bayes factor for intrinsic credibility ${\mathrm{BF}}_{\mathrm{IC}}$ similar to the definition of the *p‐*value for intrinsic credibility $p_{\mathrm{IC}}$ from (14). Intrinsic credibility at level γ is then equivalent with ${\mathrm{BF}}_{\mathrm{IC}}\leq \gamma .$ Details on the computation of ${\mathrm{BF}}_{\mathrm{IC}}$ are given in Appendix A.2. For the RECOVERY finding, the Bayes factor for intrinsic credibility is ${\mathrm{BF}}_{\mathrm{IC}}=1/25.$ This means the data favour the optimistic prior over any sceptical prior that is capable of rendering the original result no longer convincing at γ=1/25. For comparison the *p‐*value for intrinsic credibility (14) is $p_{\mathrm{IC}}=0.009.$

**FIGURE 5:**
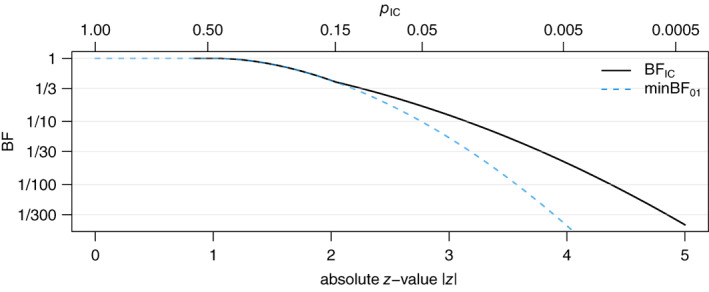
Comparison of the Bayes factor for intrinsic credibility ${\mathrm{BF}}_{\mathrm{IC}},$ the minimum Bayes factor $\;{\text{minBF}}_{01}$, and the *p*.value for intrinsic credibility $p_{\mathrm{IC}}$ as a function of the absolute *z*‐value $\left|z\right|.$ The value $p_{\mathrm{IC}}=0.15$ is at the breakpoint at $\left|z\right|=2.04$ [Colour figure can be viewed at wileyonlinelibrary.com]

Figure 5 shows the Bayes factor for intrinsic credibility ${\mathrm{BF}}_{\mathrm{IC}}$ as a function of the *z*‐value along with a comparison to the *p‐*value for intrinsic credibility $p_{\mathrm{IC}}$ and the minimum Bayes factor $\;{\text{minBF}}_{01}$ from (16). We see that the ${\mathrm{BF}}_{\mathrm{IC}}$ is undefined when $\left|z\right|<\sqrt{\log2}\approx 0.83.$ In this case the data are so unconvincing that any sceptical prior is better supported by the data than the optimistic prior. For *z*‐values between $\sqrt{\log2}\leq \left|z\right|<2.04,$ the ${\mathrm{BF}}_{\mathrm{IC}}$ equals the minimum Bayes factor $\;{\text{minBF}}_{01},$ whereas for larger *z* values $\left|z\right|\geq 2.04,$ the ${\mathrm{BF}}_{\mathrm{IC}}$ is always larger (more conservative) than the $\;{\text{minBF}}_{01}$. In the absence of any prior information, it may therefore be a useful evidential summary which formally takes into account both scepticism and optimism about the observed data.

A *p‐*value less than 0.05 is usually regarded as sufficient evidence against the null hypothesis, but how much evidence does p=0.05 mean in terms of the Bayes factor for intrinsic credibility? From Figure 5, we see that the ${\mathrm{BF}}_{\mathrm{IC}}=1/2.1$ for $\left|z\right|=1.96$, so at most “worth a bare mention” according to Jeffreys classification.^32^ Thus, also from this perspective, the conventional *p‐*value threshold of 0.05 for the claim of new discoveries seems too lax in terms of the evidential value that a finding at this threshold provides. We saw in Section 2.2 that an ordinary *p‐*value needs to be at least as small as p≤0.0056 for a finding to be intrinsically credible in terms of the *p‐*value for intrinsic credibility $p_{\mathrm{IC}}\leq 0.05.$ A *p‐*value of 0.0056 corresponds to $\left|z\right|=2.77$ where the Bayes factor for intrinsic credibility is ${\mathrm{BF}}_{\mathrm{IC}}=1/5.7,$ indicating at least “substantial” evidence against the null hypothesis according to Jeffreys. To achieve intrinsic credibility at the level for strong evidence $\left(\gamma =1/10\right)$ the requirements are even more stringent as the *z*‐value needs to be at least $\left|z\right|\geq 3.15$ (equivalent to minBF≤1/27, p≤0.002, or $p_{\mathrm{IC}}\leq 0.026).$

### 3.2 Advocacy priors

A natural question is whether we can also define an advocacy prior, a prior which renders an uncompelling finding compelling, in the AnCred framework with Bayes factors. In traditional AnCred, advocacy priors always exist since one can always find a prior that, when combined with the data, can overrule them. This is fundamentally different to inference based on Bayes factors, where the prior is not synthesised with the data, but rather used to predict them. A classical result due to Edwards et al.^54^ states that if we consider the class of all possible priors under $H_{1},$ the minimum Bayes factor is given by,

(19) $${\text{minBF}}_{01}=\exp\left\{-z^{2}/2\right\}$$

which is obtained for $H_{1}:\theta =\hat{\theta }.$ This implies that a non‐compelling finding cannot be “rescued” further than to this bound. For example, for the finding from the REMAP‐CAP trial^43^ the bound is unsatisfactorily ${\text{minBF}}_{01}=1/1.7,$ so at most “worth a bare mention” according to the classification from Jeffreys.^32^

Putting these considerations aside, we may still consider the class of $N\left(\mu ,\tau^{2}\right)$ priors under the alternative $H_{1}.$ The Bayes factor contrasting $H_{0}$ to $H_{1}$ is then given by,

$${\mathrm{BF}}_{01}=\sqrt{1+\tau^{2}/\sigma^{2}}\cdot \exp\left\{-\frac{1}{2}\left[\frac{{\hat{\theta }}^{2}}{\sigma^{2}}-\frac{{\left(\hat{\theta }-\mu \right)}^{2}}{\sigma^{2}+\tau^{2}}\right]\right\}.$$

The Reverse‐Bayes approach now determines the prior mean μ and variance $\tau^{2}$ which lead to the Bayes factor ${\mathrm{BF}}_{01}$ being just at some cut‐off γ. However, if both parameters are free, there are infinitely many solutions to ${\mathrm{BF}}_{01}=\gamma ,$ if any exist at all. The traditional AnCred framework resolves this by restricting the class of possible priors to advocacy priors with fixed coefficient of variation of $\mathrm{CV}=\tau /\mu =1/z_{\alpha /2}.$ We can translate this idea to the Bayes factor AnCred framework and fix the prior's coefficient of variation to $\mathrm{CV}=1/z\left(\gamma \right),$ where,

(20) $$z\left(\gamma \right)=\sqrt{-2\;\log\;\gamma },$$

obtained by solving (19) for z with ${\text{minBF}}_{01}=\gamma .$ The advocacy prior thus carries the same evidential weight as data with ${\text{minBF}}_{01}=\gamma$. Moreover, the determination of the prior parameters becomes more feasible since there is only one free parameter left (either μ or $\tau^{2}).$

The right plot in Figure 4 illustrates application of the procedure on data from the meta‐analysis on association between COVID‐19 mortality and corticosteroids. The coefficient of variation of the advocacy prior is fixed to $\mathrm{CV}=1/z\left(\gamma =1/3\right)=0.67$ (for comparison, the CV of the advocacy prior in traditional AnCred at α=0.05 is $\mathrm{CV}=1/z_{\alpha /2}=0.51)$ and thus the Bayes factor ${\mathrm{BF}}_{01}$ only depends on the relative mean $f=\mu /\hat{\theta }.$ Under the sceptical prior only the RECOVERY finding could be challenged at γ=1/3 (where $z\left(\gamma \right)=1.5$ corresponds to α=13%). With the advocacy prior this is now also possible for the CAPE COVID finding,^58^ where a prior with mean $\mu =f\cdot \hat{\theta }=0.37\cdot \left(-0.79\right)=-0.29$ and standard deviation τ=CV⋅μ=0.2 is able to make the finding compelling at γ=1/3. The corresponding prior credible interval for the OR at level 1−α ranges from 0.55 to 1, so advocates may still consider the “non‐compelling” finding as providing moderate evidence in favour of a benefit, if external evidence supports mortality reductions in that range. Using the prior‐to‐data conversion described in Section 2.1.3, the prior can be translated to a trial with 69 events in both arms, but 206 non‐events in the treatment and 182 non‐events in the control arm (such that the mortality rate in the control arm is the same as in the CAPE COVID trial). Note that the advocacy prior may not be unique, for example, for the CAPE COVID finding the prior with relative mean $f^{'}=1.26$ and standard deviation $\tau^{'}=0.67$ also renders the data as just compelling at γ=1/3. We recommend to choose the prior with f closer to zero, as it is the more conservative choice.

## 4 REVERSE‐BAYES ANALYSIS OF THE FALSE POSITIVE RISK

Application of the Analysis of Credibility with Bayes factors as described in Section 3 assumes some familiarity with Bayes factors as measures of evidence. Colquhoun^26^ argued that very few nonprofessional users of statistics are familiar with the notion of Bayes factors or likelihood ratios. He proposes to quantify evidence with the *false positive risk*, “if only because that is what most users still think, mistakenly, that is what the *p‐*value tells them.” More specifically, Colquhoun^26^ defines the false positive risk FPR as the posterior probability that the point null hypothesis $H_{0}$ of no effect is true given the observed *p*‐value *p*, that is, $\;\mathrm{FPR}=\mathrm{Pr}\left(H_{0}|p\right).$ As before, $H_{0}$ corresponds to the point null hypothesis $H_{0}:\theta =0.$ Note also that we take the exact (two‐sided) *p*‐value *p* as the observed “data,” regardless of whether or not it is significant at some pre‐specified level, the so‐called “*p*‐equals” interpretation of NHST.^25^

FPR can be calculated based on the Bayes factor associated with *p*. For ease of presentation we invert Bayes' theorem (1) and obtain,

(21) $$\frac{\;\mathrm{FPR}\;}{1-\mathrm{FPR}\;}=\frac{\mathrm{Pr}\left(H_{0}|p\right)}{\mathrm{Pr}\left(H_{1}|p\right)}={\mathrm{BF}}_{01}\;\frac{\mathrm{Pr}\left(H_{0}\right)}{\mathrm{Pr}\left(H_{1}\right)},$$

where $\;{\mathrm{BF}}_{01}=1/{\mathrm{BF}}_{10}$ is the Bayes factor for $H_{0}$ against $H_{1},$ computed directly from the observed *p‐*value *p*.

The common “forward‐Bayes” approach is to compute the FPR from the prior probability $\mathrm{Pr}\left(H_{0}\right)$ and the Bayes factor with (21). However, the prior probability $\mathrm{Pr}\left(H_{0}\right)$ is usually unknown in practice and often hard to assess. This can be resolved via the Reverse‐Bayes approach^25^, ^26^: Given a *p*‐value and a false positive risk value, calculate the corresponding prior probability $\mathrm{Pr}\left(H_{0}\right)$ that is needed to achieve that false positive risk. Of specific interest is the value FPR = 5%, because many scientists believe that a Type‐I error of 5% is equivalent to a FPR of 5%.^59^ This is of course not true and we follow Example 1 from Berger and Sellke^55^ and use the Reverse‐Bayes approach to derive the necessary prior assumptions on $\mathrm{Pr}\left(H_{0}\right)$ to achieve FPR = 5% with Equation (21):

(22) $$\mathrm{Pr}\left(H_{0}\right)={\left[1+\frac{1-\mathrm{FPR}\;}{\;\mathrm{FPR}\;}\cdot {\mathrm{BF}}_{01}\right]}^{-1}.$$

Colquhoun^25^ uses a Bayes factor based on the *t*‐test, but for compatibility with the previous sections we assume normality of the underlying test statistic. We consider Bayes factors under all simple alternatives, but also Bayes factors under local normal priors, see Held and Ott^51^ for a detailed comparison.

Instead of working with a Bayes factor for a specific prior distribution, we prefer to work with the minimum Bayes factor $\;{\text{minBF}}_{01}$ as introduced in Section 3.1. In what follows we will use the minimum Bayes factor based on the z‐test, see Section 2.1 and 2.2 in Held and Ott.^51^

Let $\;{\text{minBF}}_{01}$ denote the minimum Bayes factor over a specific class of alternatives. From Equation (22) we obtain the inequality

(23) $$\mathrm{Pr}\left(H_{0}\right)\leq {\left[1+\frac{1-\mathrm{FPR}\;}{\;\mathrm{FPR}\;}\cdot {\text{minBF}}_{01}\right]}^{-1}.$$

The right‐hand side is thus an upper bound on the prior probability $\mathrm{Pr}\left(H_{0}\right)$ for a given *p‐*value to achieve a pre‐specified FPR value.

There are also minBFs not based on the *z*‐test statistic as (16), but directly on the (two‐sided) *p*‐value *p*, the so‐called “−eplogp” ^56^ calibration,

(24) $$\;\text{minBF}=\left\{\begin{array}{ll} -e\;p\;\log\;p & \;\text{for}\;p<1/e\\ 1 & \;\text{otherwise},\; \end{array}\right.$$

and the “−eqlogq” calibration, where q=1−p, see Section  2.3 in Held and Ott^51^:

(25) $$\;\text{minBF}=\left\{\begin{array}{ll} -e\;\left(1-p\right)\log\left(1-p\right) & \;\text{for}\;p<1-1/e\\ 1 & \;\text{otherwise}.\; \end{array}\right.$$

For small *p*, Equation (25) can be simplified to minBF≈ep, which mimics the Good^60^ transformation of *p‐*values to Bayes factors.^61^

The two *p*‐based calibrations carry less assumptions than the minimum Bayes factors based on the *z*‐test under normality and can be used as alternative expressions in (23). The “−eplogp” provides a general bound under all unimodal and symmetrical local priors for *p*‐values from *z*‐tests, see Section 3.2 in Sellke et al.^56^ The “−eqlogq” calibration is more conservative and gives a smaller bound on the Bayes factor than the “−eplogp” calibration. It can be viewed as a general lower bound under simple alternatives where the direction of the effect is taken into account, see Sections 2.1 and 2.3 in Held and Ott.^51^

**FIGURE 6:**
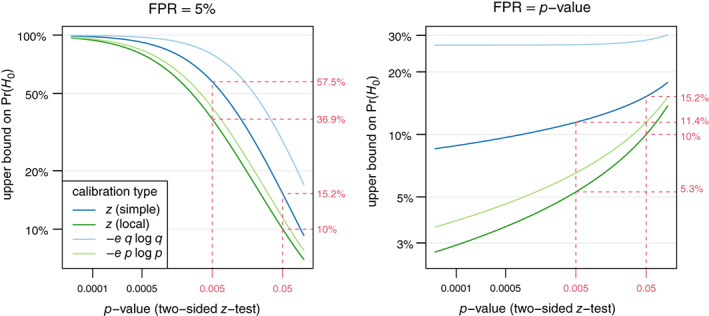
The left plot shows the upper bound on the prior probability $\mathrm{Pr}\left(H_{0}\right)$ to achieve a false positive risk of 5% as a function of the *p*‐value calibrated with either a *z*‐test calibration (simple or local alternatives) or with the "−eplogp" or "−eqlogq" calibrations, respectively. The right plot shows the upper bound on $\mathrm{Pr}\left(H_{0}\right)$ as a function of the *p‐*value using the same calibrations but assuming the *p*‐value equals the FPR [Colour figure can be viewed at wileyonlinelibrary.com]

The left plot in Figure 6 shows the resulting upper bound on the prior probability $\mathrm{Pr}\left(H_{0}\right)$ as a function of the two‐sided *p*‐value if the FPR is fixed at 5%. For p=0.05, the “−eplogp” bound is around 11% and 28% for the “−eqlogq” calibration. The corresponding values based on the *z*‐test are slightly smaller (10% and 15%, respectively). All the probabilities are below the 50% value of equipoise, illustrating that borderline significant result with p≈0.05 do not provide sufficient evidence to justify an FPR value of 5%. For p=0.005, the upper bounds are closer to 50% (37% for local and 57% for simple alternatives).

Turning again to the example from the RECOVERY trial,^36^ the *p‐*value associated with the estimated treatment effect is p=0.0002. The left plot in Figure 6 shows that the false positive risk can safely be assumed to be around 5% (or lower), since the upper bound on $\mathrm{Pr}\left(H_{0}\right)$ are all very large for such a small *p‐*value. Fixing FPR at the 5% level may be considered as arbitrary. Another widespread misconception is the belief that the FPR is equal to the *p‐*value. Held^24^ used a Reverse‐Bayes approach to investigate which prior assumptions are required such that FPR=p holds. Combining (22) with the “−eplogp” calibration (24) gives the explicit condition,

$$\mathrm{Pr}\left(H_{0}\right)\leq 1/\left\{1-e\;\left(1-p\right)\;\log\left(p\right)\right\}$$

whereas the “−eqlogq” calibration (25) leads to,

$$\mathrm{Pr}\left(H_{0}\right)\leq 1/\left\{1-e\;\frac{{\left(1-p\right)}^{2}}{p}\;\log\left(1-p\right)\right\}\approx 1/\left\{1+e\;\left(1-p\right)\right\},$$

which is approximately $1/\left(1+e\right)=26.9$% for small p.

The right plot in Figure 6 compares the bounds based on these two calibrations with the ones obtained from simple respectively local alternatives. We can see that strong assumptions on $\mathrm{Pr}\left(H_{0}\right)$ are needed to justify the claim $\;\mathrm{FPR}=p:\mathrm{Pr}\left(H_{0}\right)$ cannot be larger than 15.2% if the *p‐*value is conventionally significant (*p* < 0.05). For p<0.005, the bound drops further to 11.4%. Even under the conservative “−eqlogq” calibration, the upper bound on $\mathrm{Pr}\left(H_{0}\right)$ is 26.9% for small p and increases only slightly for larger values of p. This illustrates that the misinterpretation FPR=p only holds if the prior probability of $H_{0}$ is substantially smaller than 50%, an assumption which is questionable in the absence of strong external knowledge.

## 5 DISCUSSION

### 5.1 Extensions, work in progress and outlook

The Reverse‐Bayes methods described above have focused on the comparison of the prior needed for credibility with findings from other studies and/or more general insights. However, replication studies make an obvious additional source of external evidence, as these are typically conducted to confirm original findings by repeating their experiments as closely as possible. The question is then whether the original findings have been successfully “replicated,” currently of considerable concern to the research community. To date, there remains no consensus on the precise meaning of replication in a statistical sense. The proposal of Held^28^ (see also Held et al.^62^) was to challenge the original finding using AnCred, as described in Section 2.1, and then evaluate the plausibility of the resulting prior using a prior‐predictive check on the data from a replication study. A similar procedure but using AnCred based on Bayes factors as in Section 3 was proposed in Pawel and Held.^29^ Reverse‐Bayes inference seems to fit naturally into this setting as it provides a formal framework to challenge and substantiate scientific findings.

Apart from using data from a replication study, there are also other possible extensions of AnCred: We proposed to derive Reverse‐Bayes priors using posterior tail probabilities (or credible intervals) or Bayes factors as measures of evidence, but also other measures such as relative belief ratios^63^ could be used. When testing point null hypotheses, relative belief ratios are equivalent to Bayes factors due to the Savage‐Dickey density ratio.^63^ ^(p98)^ Therefore, determining the sceptical prior variance through fixing the resulting Bayes factor is equivalent to fixing the resulting relative belief ratio. However, there is no connection to relative belief in prior‐data conflict assessment based on the Bayes factor contrasting the sceptical to the optimistic prior since both are composite. Further research is needed on Reverse‐Bayes procedures in the relative belief framework, candidate methods for prior‐data conflict assessment are prior to posterior divergence^13^ and prior expansions^14^ as these methods have an interpretation in terms of relative beliefs. Moreover, we either used prior‐predictive checks^11^, ^12^ or Bayes‐factors^32^, ^33^ for the formal evaluation of the plausibility of the priors derived through Reverse‐Bayes. Other methods could be used for this purpose, for example, Bayesian measures of surprise.^64^ Furthermore, AnCred in its current state is derived assuming a normal likelihood for the effect estimate $\hat{\theta }$. This is the same framework as in standard meta‐analysis and provides a good approximation for studies with reasonable sample size.^65^ For the comparison of binomial outcomes with small counts, the normal approximation of the log‐odds ratio could be improved with a Yates continuity correction^21^ ^(sec2.4.1)^ or replaced with the exact profile likelihood of the log‐odds ratio,^66^ ^(sec5.3)^ see also Section 4 in Pawel and Held^29^ which shows AnCred with Bayes factors using either a non‐central t or a binomial likelihood. Likewise, the conjugate normal prior could be replaced by a more robust prior distribution such as a mixture of normals (as considered in Section 2.1.2), a double‐exponential, or a Student *t*‐distribution.^67^ For example, Fúquene^68^ investigate the use of robust priors in an application to binomial data from a randomised controlled trial. In general, any distribution from the location‐scale family can be used, whereby the scale parameter takes over the role of the sceptical prior standard deviation, while the location parameter is fixed to the null value.

### 5.2 Conclusions

The inferential advantages of Bayesian methods are increasingly recognised within the statistical community. However, among the majority of working researchers they have failed to make any serious headway, and retain a reputation for complex and “controversial.” We have outlined how an idea that began with Jack Good's proposal for resolving the “Problem of Priors” over 70 years ago^10^ has experienced a renaissance over recent years. The basic idea is to invert Bayes' theorem: a specified posterior is combined with the data to obtain the Reverse‐Bayes prior, which is then used for further inference. This approach is useful in situations where it is difficult to decide what constitutes a reasonable prior, but easy to specify the posterior which would lead to a particular decision. A subsequent prior‐to‐data conversion^22^ helps to assess the weight of the Reverse‐Bayes prior in relation to the actual data.

We have shown that the Reverse‐Bayes methodology is useful to extract more insights from the results typically reported in a meta‐analysis. It facilitates the computation of prior‐predictive checks for conflict diagnostics^35^ and has been shown capable of addressing many common inferential challenges, including assessing the credibility of scientific findings,^21^, ^23^ making sense of “out of the blue” discoveries with no prior support,^27^, ^41^ estimating the probability of successful replications,^27^, ^28^ and extracting more insight from standard *p‐*values while reducing the risk of misinterpretation.^24^, ^25^, ^26^ The appeal of Reverse‐Bayes techniques has recently been widened by the development of inferential methods using both posterior probabilities and Bayes factors.^18^, ^29^

These developments come at a crucial time for the role of statistical methods in research. Despite the many serious—and now well‐publicised—inadequacies of NHST,^3^ the research community has shown itself to be remarkably reluctant to abandon NHST. Techniques based on the Reverse‐Bayes methodology of the kind described in this review could encourage the wider use of Bayesian inference by researchers. As such, we believe they can play a key role in the scientific enterprise of the 21th century.

## CONFLICT OF INTEREST

The authors declare no potential conflict of interests.

## Data Availability

All analyses were performed in the R programming language version 4.1.2.^69^ Minimum Bayes factors were computed using the package pCalibrate.^51^ The package metafor ^48^ was used for meta‐analysis and forest plots. Data and code to reproduce all analyses are available at https://gitlab.uzh.ch/samuel.pawel/Reverse-Bayes-Code.

## A.1. Mean of the advocacy prior

Suppose that the estimate $\hat{\theta }$ is not significant at level α, so $z^{2}/z_{\alpha /2}^{2}<1.$ With $U,L=\hat{\theta }\pm z_{\alpha /2}\;\sigma$ we have $U+L=2\;\hat{\theta },$ $\mathrm{UL}={\hat{\theta }}^{2}-z_{\alpha /2}^{2}\;\sigma^{2}$ and $U-L=2\;z_{\alpha /2}\;\sigma$.

We therefore obtain with (11):

$$\mu =\frac{\;\mathrm{AL}\;}{2}=-\frac{2\;\hat{\theta }}{2\;\left({\hat{\theta }}^{2}-z_{\alpha /2}^{2}\;\sigma^{2}\right)}\frac{{\left(2\;z_{\alpha /2}\;\sigma \right)}^{2}}{2}=\frac{2\;\hat{\theta }\;z_{\alpha /2}^{2}\;\sigma^{2}}{z_{\alpha /2}^{2}\;\sigma^{2}-{\hat{\theta }}^{2}}=\frac{2\;\hat{\theta }}{1-z^{2}/z_{\alpha /2}^{2}}.$$

Dividing by the effect estimate $\hat{\theta }$ leads to the relative mean $f=\mu /\hat{\theta }$ as in (12). The advocacy standard deviation is $\tau =\mathrm{AL}/\left(2\;z_{\alpha /2}\right)=\mu /z_{\alpha /2}$ and the coefficient of variation is therefore $\;\mathrm{CV}=\tau /\mu =z_{\alpha /2}^{-1}.$

## A.2. Bayes factor for intrinsic credibility

Intrinsic credibility at level γ is established when,

(A1)
$${\mathrm{BF}}_{12}\leq {\mathrm{BF}}_{01}=\gamma$$

and we are interested in the Bayes factor for intrinsic credibility ${\mathrm{BF}}_{\mathrm{IC}}$ which is the smallest level $\gamma \in \left(0,1\right]$ where (A1) holds. The ${\mathrm{BF}}_{\mathrm{IC}}$ is therefore a special case of the sceptical Bayes factor from Pawel and Held^29^ where the same data is used in both Bayes factors (instead of the data from a replication study for ${\mathrm{BF}}_{12}).$ It is hence given by,

(A2)
$${\mathrm{BF}}_{\mathrm{IC}}=\left\{\begin{array}{ll} \frac{\sqrt{-z^{2}/k}}{\exp\left\{\left(z^{2}+k\right)/2\right\}} & \text{if}\;\left|z\right|\geq J\\ \;{\text{minBF}}_{01} & \text{if}\;\sqrt{\log2}\leq \left|z\right|<J\\ \text{undefined} & \text{if }\left|z\right|<\sqrt{\log2} \end{array}\right.$$

with $k=W\left(-z^{2}\exp\left\{-z^{2}/2\right\}/\sqrt{2}\right),\;J\;=\sqrt{-2W\left(-e^{-1}/\sqrt{2}\right)}$, and $W\left(\cdot \right)$ the branch of the Lambert W function that satisfies $W\left(y\right)\leq -1$ for $y\in \left[-e^{-1},0\right).$ If $\left|z\right|\geq J\approx 2.04,$ ${\mathrm{BF}}_{\mathrm{IC}}$ is located at the intersection between ${\mathrm{BF}}_{12}$ and ${\mathrm{BF}}_{01}$ in the relative prior variance *g*, so Equation (7) from Pawel and Held^29^ can be used. For $\left|z\right|\geq \sqrt{\log2}\approx 0.83,$ ${\mathrm{BF}}_{12}$ remains below ${\mathrm{BF}}_{01}$ for all g and hence ${\mathrm{BF}}_{\mathrm{IC}}$ is given by $\;{\text{minBF}}_{01},$ the minimum of ${\mathrm{BF}}_{01}$ from (16). Finally, when $z<\sqrt{\log2}$, Equation (A1) cannot be satisfied for any valid sufficiently sceptical relative prior variance g, hence the ${\mathrm{BF}}_{\mathrm{IC}}$ is undefined.
